# Benefits for children with suspected cancer from routine whole-genome sequencing

**DOI:** 10.1038/s41591-024-03056-w

**Published:** 2024-07-02

**Authors:** Angus Hodder, Sarah M. Leiter, Jonathan Kennedy, Dilys Addy, Munaza Ahmed, Thankamma Ajithkumar, Kieren Allinson, Phil Ancliff, Shivani Bailey, Gemma Barnard, G. A. Amos Burke, Charlotte Burns, Julian Cano-Flanagan, Jane Chalker, Nicholas Coleman, Danny Cheng, Yasmin Clinch, Caryl Dryden, Sara Ghorashian, Blanche Griffin, Gail Horan, Michael Hubank, Phillippa May, Joanna McDerra, Rajvi Nagrecha, James Nicholson, David O’Connor, Vesna Pavasovic, Annelies Quaegebeur, Anupama Rao, Thomas Roberts, Sujith Samarasinghe, Iryna Stasevich, John A. Tadross, Claire Trayers, Jamie Trotman, Ajay Vora, James Watkins, Lyn S. Chitty, Sarah Bowdin, Ruth Armstrong, Matthew J. Murray, Catherine E. Hook, Patrick Tarpey, Aditi Vedi, Jack Bartram, Sam Behjati

**Affiliations:** 1 Great Ormond Street Hospital NHS Foundation Trust, London, UK; 2 Wellcome Sanger Institute, Hinxton, UK; 3 Cambridge University Hospitals NHS Foundation Trust, Cambridge, UK; 4 Department of Paediatrics, University of Cambridge, Cambridge, UK; 5 North Thames Genomic Laboratory Hub, London, UK; 6 Department of Pathology, University of Cambridge, Cambridge, UK; 7 UCL Great Ormond Street Institute of Child Health, London, UK; 8 The Institute of Cancer Research, London, UK; 9 UCL Cancer Institute, University College London, London, UK; 10 Department of Clinical Neurosciences, University of Cambridge, Cambridge, UK; 11 East Genomics Laboratory Hub, Cambridge, UK; 12 MRC Metabolic Diseases Unit, Wellcome Trust-Medical Research Council Institute of Metabolic Science, University of Cambridge, Cambridge, UK

**Keywords:** Paediatrics, Genomics, Cancer genomics, Genetics research

## Abstract

Clinical whole-genome sequencing (WGS) has been shown to deliver potential benefits to children with cancer and to alter treatment in high-risk patient groups. It remains unknown whether offering WGS to every child with suspected cancer can change patient management. We collected WGS variant calls and clinical and diagnostic information from 281 children (282 tumors) across two English units (*n* = 152 from a hematology center, *n* = 130 from a solid tumor center) where WGS had become a routine test. Our key finding was that variants uniquely attributable to WGS changed the management in ~7% (20 out of 282) of cases while providing additional disease-relevant findings, beyond standard-of-care molecular tests, in 108 instances for 83 (29%) cases. Furthermore, WGS faithfully reproduced every standard-of-care molecular test (*n* = 738) and revealed several previously unknown genomic features of childhood tumors. We show that WGS can be delivered as part of routine clinical care to children with suspected cancer and can change clinical management by delivering unexpected genomic insights. Our experience portrays WGS as a clinically impactful assay for routine practice, providing opportunities for assay consolidation and for delivery of molecularly informed patient care.

## Abstract (web summary)

In a cohort of 281 children with diagnosed or suspected cancer presenting to the NHS, implementing routine whole-genome sequencing provided clinical benefit in 29% of cases and led to change in management in 7% of patients.

## Main

Efforts of the past decade have defined variants that underpin human cancer. As DNA sequencing and analysis have become more easily accessible and less costly, it is being adopted into routine oncological practice^1,2^. A variety of assays are available to clinicians, with whole-genome sequencing (WGS) representing the most informative singular assay, providing a readout of all classes of variants across the entire (accessible) genome^3–5^.

Studies exploring the clinical benefits of cancer WGS indicate that it is particularly fruitful in pediatric oncological practice, perhaps because childhood cancer treatment is often guided by genetic features^6^. Furthermore, the genetic basis of some types of childhood cancer are relatively unexplored, increasing the chances of revealing clinically valuable variants. Research exploring the benefits of WGS suggests that it can provide additional relevant information even when standard-of-care (SOC) testing includes expansive molecular assays such as targeted DNA and RNA sequencing panels^7–10^.

Given this potential, WGS has been incorporated into clinical practice through different service models^11^. In pediatric oncology, for example, cancer WGS is often delivered by supra-regional centers in isolation, which have usually evolved from previous academic cancer genomics efforts. In this setting, WGS is commonly deployed for select patient groups, in particular, for children with high-risk tumors and relapsed disease, combined with other sequencing modalities^12–18^. By contrast, some countries have implemented national initiatives for WGS, such as the Genomic Medicine Service established within the National Health Service (NHS) in England^19^, the Australian Zero Childhood Cancer Program^20^ and the Swedish GMS Childhood Cancer project^21,22^.

Despite the increasing adoption of WGS, there is a paucity of evidence supporting the routine use of cancer WGS in pediatric practice. Past studies providing evidence of utility have largely focused on select patient groups such as children with high-risk disease^12–18^ or examined nonconsecutive cohorts of non-high-risk patients^21,22^. Furthermore, the benefits of WGS have mostly been studied as potential benefits and the real-time impact of WGS information on patient care has not been assessed^19,21–25^. As such, it remains unknown whether WGS incorporated into routine clinical practice can provide clinical benefits to all children with suspected cancer beyond SOC molecular tests.

**Fig. 1:**
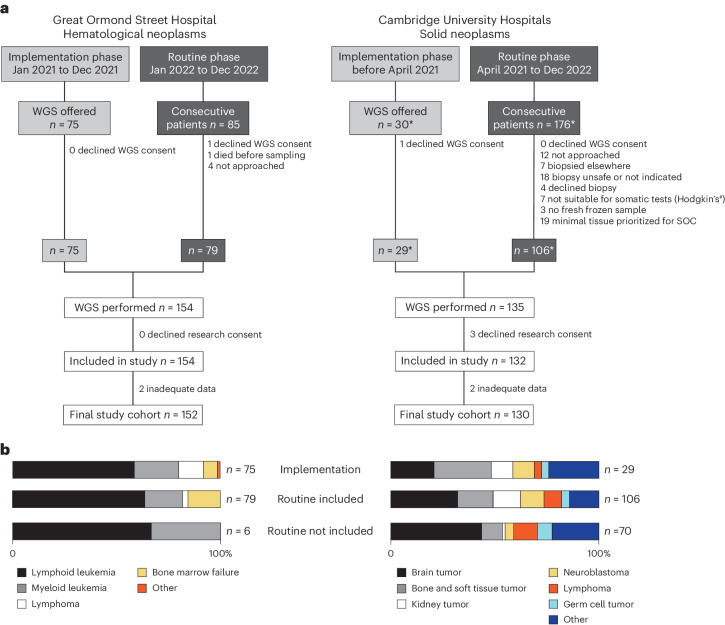
Study recruitment. **a**,**b**, Recruitment of patients was split by site and study phase (**a**) and by tumor type (**b**). The patients were recruited from two sites where WGS became routine. The octothorpe indicates that a low tumor cell fraction in Hodgkin’s disease precludes any somatic molecular test (be it SOC or WGS). The asterisks indicate that one patient (CUH_0034) underwent WGS twice: at diagnosis during the implementation phase and at relapse during the routine phase (Supplementary Table 1).

**Fig. 2:**
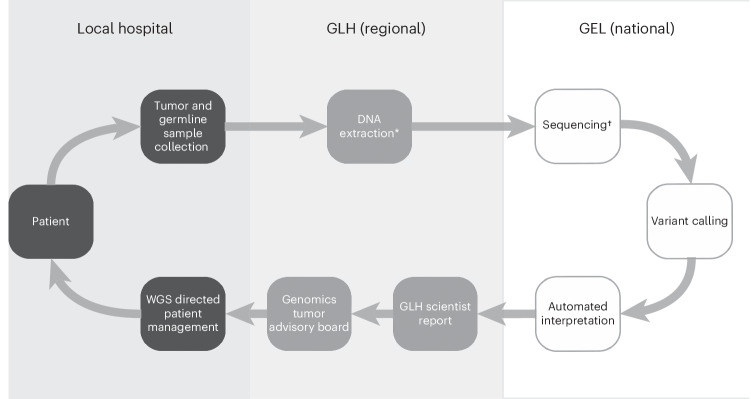
NHS England WGS centralized workflow. There are seven regional GLHs in England. Genomics England is a private company owned by the UK government, responsible for WGS, variant calling and annotation. GLH scientists pull this information from a centralized portal in the form of an automated WGS report (with pertinent variants stratified by potential actionability), interpreting results within their clinical context, before cases are brought to a GTAB. GTABs include, at a minimum, reporting scientists, clinical geneticists and treating clinicians. GTAB reports are made available as a part of patient notes, informing clinical practice. The asterisk indicates that DNA extraction is performed in GLHs and is submitted for centralized WGS via a centralized plating service located elsewhere. The dagger indicates that sequencing is carried out in a central facility to a depth of approximately 100× for tumors and 40× for germlines.

This question may be answered through the NHS WGS service that, in principle, offers WGS to every child with a suspected neoplastic disorder in England. Established in January 2021 and built on the infrastructure of the NHS England 100,000 Genomes Project^19^, the program offers WGS sequencing and analyses through a national pipeline, returning variant calls to clinicians for local, personalized decision-making. Uptake of WGS across English pediatric oncological units varies, thus precluding a national study into the clinical utility of routine delivery. However, two units in England—Great Ormond Street Hospital (GOSH), London, and Cambridge University Hospitals (CUH)—systematically deployed WGS for consecutive children with leukemia and solid tumors, respectively (Figs. 1 and 2). This has enabled us to evaluate the benefits of routine WGS on clinical practice in an observational study, which we report here.

## Results

### Overview of the study cohort

During the implementation phase (Methods), children were offered WGS at the discretion of their treating clinician. During the routine testing phase, we aimed to offer WGS to consecutive children at both sites, recruiting 93% and 90% of eligible children at GOSH and CUH, respectively. There was near-universal uptake (289 out of 291) by children and/or their legal guardians. WGS data were successfully generated from all, bar four, specimens. Three families declined research consent. Overall, we examined 282 tumors from 281 children, as detailed in Fig. 1, of whom 19 (7%) presented with relapsed disease. Turnaround times, from test request to results being available for clinical decision-making, were variable and decreased over time, with a median duration of 18 days (range = 9 to 64 days) for solid tumors and 19 days (range = 11 to 71 days) for hematological malignancies. The spectrum of tumors encompassed 75 entities that were broadly representative of pediatric oncological (including lymphomas and histiocytoses) and hematological practice. Children with neoplasms of the axial skeleton and primary bone tumors were relatively underrepresented. Due to national service configurations in England, these children mostly underwent biopsies at separate regional sarcoma units outside the ethical remit of our study. A second underrepresented group was composed of children with low-grade brain tumors who, following surgery, typically have infrequent hospital appointments, providing oncologists with few opportunities for offering WGS. Among children with hematological malignancies, we did not capture adolescents (children older than 12 years) as these are not cared for at GOSH owing to local service configurations.

### Comparison to SOC molecular testing

**Fig. 3:**
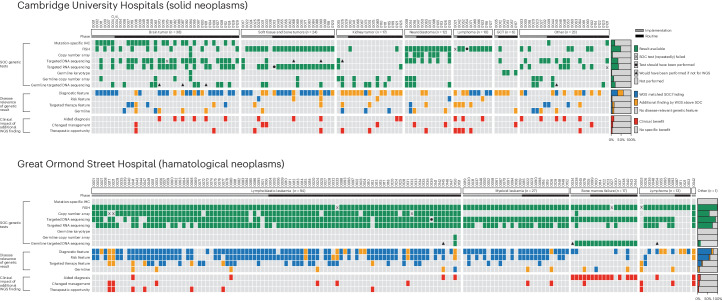
WGS findings compared with SOC genomic test results, split by study site. Each case shows which SOC tests were performed (green if successful, ‘X’ if failed). We highlight where tests should have been performed, or would have, had it not been for WGS (as defined in Extended Data Table 1). Where genomic findings provided disease-relevant information, we highlight cases where WGS matched SOC (blue) or provided additional information (yellow). There were no cases in which WGS did not match SOC. Definitions of benefit and numbers in each category can be found in Table 1. A full case-by-case breakdown of all SOC and WGS findings can be found in Extended Data Table 1. For mutation-specific IHC, the following three mutations were included: *BRAF* V600E, *IDH1* R132H and *H3F3A* K27M. GCT, germ cell tumors.

SOC tumor and germline molecular tests (not counting epigenetic assays) deployed in our study cohort included copy number arrays (tumor, germline or both), fluorescence in situ hybridization (FISH, tumor), mutation-specific immunohistochemistry (IHC; tumor), targeted DNA sequencing (tumor, germline or both), targeted RNA sequencing for gene fusion detection (tumor) and karyotyping (germline). Their use across the cohort, and within specific entities, was variable (Fig. 3 and Supplementary Table 1), reflecting routine pediatric cancer care (Extended Data Tables 1 and 2).

For example, the SOC molecular testing deployed for leukemia in this cohort comprised FISH and targeted DNA and RNA sequencing in most cases, whereas lymphomas were typically interrogated using only FISH at CUH. Across the overall cohort, 738 SOC molecular tests were deployed. WGS faithfully reproduced findings (including absence of disease-relevant features) from all these assays and revealed additional diagnostic, risk, therapeutic and germline features in 108 instances (83 out of 282 cases, 29%), of which 80 features added clinical benefits (Fig. 3 and Tables 1 and 2). WGS findings and their benefits are presented for each child in Supplementary Table 1.

**Table 1:** Definitions of WGS impact categories

Impact	Category	Definition	Proportion
Additional disease-relevant findings 108 instances from 83 cases (29%)	Diagnostic	Makes, clarifies or refines the diagnosis	20% (*n* = 55)
	Risk category	Places malignancy into a formal risk category as per trial protocol or treatment guideline used for patient management	4% (*n* = 12)
	Mutation target	Makes eligible for specific medication to be used during treatment or relapse; must be feasible to obtain drug (for example, through open trial or precedent of compassionate use)	7% (*n* = 20)
	Pathogenic germline variant	Identification of a cancer predisposition variant; either expected (new, expected), for example, because of clinical suspicion, or unexpected (new), when there was no clinical suspicion or indication to perform germline screening	7% (*n* = 21)
Clinical benefit 80 instances from 69 cases (24%)	Aided diagnosis	Accelerated genetic findings or additional diagnostic information that led to child having a modified or clarified diagnosis without substantive change to management	14% (*n* = 40)
	Therapeutic opportunity	WGS findings made child eligible for mutation-targeting medication during treatment and at relapse, but it was not given	7% (*n* = 20)
	Changed management	Unique WGS finding that changed clinical care; finding was not obtained through SOC testing and would or should not have been looked for	7% (*n* = 20)

### Clinical benefits

WGS provided clinical benefit in 80 instances across 69 out of 282 (24%) cases in the following three domains: aiding diagnoses (*n* = 40), through accelerated expected findings, or additional diagnostic information; providing therapeutic opportunities (*n* = 20) such as variants targetable through existing drugs; and changing management (*n* = 20), as discussed in detail below. A summary of impacts is provided in Table 2, and case-level information is detailed in Supplementary Table 1. A disease spectrum that particularly benefited from WGS as a diagnostic tool was nonneoplastic bone marrow failure (*n* = 17 children). Children with bone marrow failure undergo extensive genetic testing (including up to four different panels of targeted gene sequencing), with some assays having lengthy nationally agreed turnaround times in the English healthcare system (for example, 90 days). In this specific context, WGS provided all necessary genetic results comparably fast (median = 17 days) in a singular test, thus leading to accelerated definitive therapy, which is likely to improve outcomes by reducing pretreatment morbidity and mortality^26–28^.

**Table 2:** Clinical benefits from WGS

Type of clinical benefit	All cases (*n* = 282)	Solid tumor cohort (*n* = 130)		Hematology cohort (*n* = 152)	
		Implementation	Routine	Implementation	Routine
Aided diagnosis: *n* = 40 (14%)					
Accelerated diagnosis^a^	24	1	6	6	11
Other new diagnostic information^b^	16	3	9	3	1
Therapeutic opportunity: *n* = 20 (7%)					
Mutation-guided treatment option^c^	20	3	9	7	1
Actual change in management: *n* = 20 (7%)					
Clarified diagnosis	2	1	1	0	0
Clarified risk	5	0	0	3	2
Predisposition cascade testing ± secondary cancer screening	11	1	3	4	3
Predisposition-directed therapy	2	1	1	0	0

^a^Accelerated diagnoses were provided across the following domains: bone marrow failure (*n* = 14), expected cancer predisposition (*n* = 7), ruled out cancer predisposition (*n* = 1), identification of risk-defining variant (*n* = 2).

^b^Other new diagnostic information was provided across the following domains: clarified diagnosis (*n* = 8), delineated a distinct entity (*n* = 3), clarified diagnostic discrepancies in SOC molecular tests (*n* = 2), clarified risk category (*n* = 2), minimal residual disease marker that was actually used (*n* = 1).

^c^Treatment opportunity for checkpoint inhibitors (*n* = 4), RAS pathway inhibitors (*n* = 8) and others (*n* = 8).

### Unique WGS findings that changed management

WGS delivered findings that changed management in 20 children overall, 10 from the implementation phase and 10 from the routine testing phase (Table 3). The children benefiting from WGS had a variety of (non-relapsed) diseases that would mostly be considered standard risk within their respective diagnostic category. In identifying these cases, we applied stringent criteria (Methods) to identify only those children in whom SOC assays would not have delivered the management-changing finding. For example, WGS revealed a breast-cancer-predisposing germline variant (frameshift variant in *PALB2*) in a girl with a thoracic neuroblastoma (CUH_0016). This discovery led to a change in her radiotherapy field to avoid exposure of breast buds. Although this child had undergone SOC germline molecular testing (karyotyping, copy number array, direct sequencing of neuroblastoma predisposition genes), *PALB2* had not been interrogated as it is viewed as irrelevant in neuroblastoma predisposition investigations. Accordingly, we considered that WGS led to a treatment change in this child through a finding that had not been found, and would not have been looked for, by SOC germline or tumor assays. A hematological example was the discovery of a somatic *IGH–DUX4* gene fusion in a child with B cell acute lymphoblastic leukemia (GOS_0131) that, due to variable and repetitive sequences^29^, is rarely detected by SOC assays (that is, FISH, targeted RNA sequencing and copy number array)^7,10^. Ordinarily, the child would have been offered intensified therapy because of residual disease. However, the *IGH–DUX4* gene fusion is known to render leukemias slowly responding, yet confers good overall prognosis^10,30,31^. This WGS finding therefore enabled clinicians not to escalate therapy. Following this detailed and nuanced approach to case review, the 20 instances of management-changing WGS findings we identified fell into the categories detailed in Table 3, with germline variants featuring prominently.

**Table 3:** Management-changing WGS findings

Diagnosis (patient)	Implementation (i) or routine (r) phase	First presentation (1st) or relapse (Rel)	Disease-specific risk group	Vignette
Discovery of unexpected predisposition that led to a change in treatment				
Thoracic neuroblastoma (CUH_0016)	i	1st	High risk	WGS identified breast cancer predisposition (*PALB2* variant), which led to a change of the irradiation field to avoid breast buds.
Wilms tumor (CUH_0080)	r	1st	Non-high risk	WGS identified two *TRIM28* variants that appeared to be somatic (that is, absent from blood DNA). Because *TRIM28* variants usually arise in the context of germline or mosaic predisposition, this finding led to testing of normal kidney tissue where a mosaic *TRIM28* variant was found, based on which the child was treated on a chemotherapy schedule for predisposed children.
WGS findings affecting risk category that led to a change in management				
Mixed-phenotype acute leukemia (GOS_0042)	i	1st	High risk	SOC molecular tests (including panel RNA sequencing) did not find a risk-defining mutation. WGS revealed a *KMT2A-USP2* fusion that defined this disease as high risk, leading to the patient receiving an allogeneic transplant.
B cell acute lymphoblastic leukemia (GOS_0131)	r	1st	Non-high risk	Incomplete treatment response, which would have led to treatment escalation. WGS identified an *IGH–DUX4* fusion (known to be undetectable by SOC molecular tests including targeted RNA sequencing), which defines a variant of non-high-risk disease with slow-response kinetics, meaning that treatment was not escalated.
B cell acute lymphoblastic leukemia (GOS_0017)	i	1st	Non-high risk	Failure of SOC molecular tests due to poor-quality samples (including a targeted RNA panel from a trephine, which failed to detect an *ETV6–RUNX1* lesion). DNA was, however, of sufficient quality to generate adequate WGS data that showed good risk cytogenetics (high hyperdiploidy or *ETV6–RUNX1* fusion) enabling treatment de-escalation.
B cell acute lymphoblastic leukemia (GOS_0021)	i	1st	Non-high risk	
B cell acute lymphoblastic leukemia (GOS_0033)	r	1st	Non-high risk	
WGS finding affecting diagnosis that led to a change in management				
Rhabdomyosarcoma (CUH_0033)	i	1st	Non-high risk	Challenging diagnosis by conventional diagnostic workup including SOC molecular tests with the main differential diagnoses being rhabdomyosarcoma and pleuropulmonary blastoma. WGS revealed features consistent with the former while showing the absence of variants supporting the latter. These WGS findings thus led to treating this child with rhabdomyosarcoma maintenance therapy.
Myofibroma (CUH_0102)	r	1st	Non-high risk	Histologically ambiguous case in which WGS identified a *PDGFRB* variant that, in the context of the clinical picture and histology, was diagnostic of myofibroma enabling treatment as benign tumor.
Discovery of unexpected predisposition that led to cascade testing and/or cancer screening—related to diagnosis				
Malignant peripheral nerve sheath tumor (CUH_0031)	i	1st	Non-high risk	Identification of unexpected cancer predisposition (*DICER1*)
B cell acute lymphoblastic leukemia (GOS_0024)	r	1st	Non-high risk	Identification of unexpected cancer predisposition (*TP53*)
Neuroblastoma (CUH_0126)	r	1st	Non-high risk	Identification of unexpected cancer predisposition (bonafide neuroblastoma predisposition not further specified)
Discovery of unexpected predisposition that led to cascade testing and/or cancer screening—conventionally not thought to be related to diagnosis				
High-grade glioma (CUH_0038)	r	1st	High risk	Identification of unexpected cancer predisposition (*MSH6*)
T cell lymphoblastic lymphoma (GOS_0003)	i	1st	Non-high risk	Identification of unexpected cancer predisposition (*PMS2*)
Yolk sac tumor (CUH_0117)	r	1st	Non-high risk	Identification of unexpected cancer predisposition (*FLCN*)
B cell acute lymphoblastic leukemia (GOS_0053)	i	1st	Non-high risk	Identification of unexpected adult cancer predisposition (*CHEK2*)
Aplastic anemia (GOS_0079)	i	1st	Non-high risk	Identification of unexpected cancer predisposition (*PMS2*)
High-grade B cell non-Hodgkin lymphoma (GOS_0092)	i	1st	Non-high risk	Identification of unexpected adult cancer predisposition (*BRCA2*)
Acute myeloid leukemia (GOS_0126)	r	1st	Non-high risk	Identification of unexpected adult cancer predisposition (*PALB2*)
Acute myeloid leukemia (GOS_0022)	r	1st	High risk	Identification of unexpected cancer predisposition (*MUTYH*; considered significant in this child because of family history)

### Novel features of the childhood cancer genome

In some cases (*n* = 6), WGS defined previously unknown genetic features that may lend themselves to further investigation (Table 4), encompassing distinct molecular tumor entities, disease-defining mutations and variations of established cancer-causing mutations. An example of a distinct tumor entity was an infant high-grade brain tumor with an *ATNX1–NUTM2D* gene fusion not previously described in children. The cancer had been unclassifiable by histology and by SOC molecular assays, including methylation profiling by array, which identified this tumor as unique among tens of thousands of reference points. An instance of a disease-defining mutation was a *FOSL1* rearrangement in a fibroma-like tumor that we established in a separate study as pathognomonic of desmoplastic fibroblastoma^32^.

**Table 4:** Novel features of the childhood cancer genome

Diagnosis (patient)	Research finding detail
Desmoplastic fibroblastoma (CUH_0048)	*FOSL1* as a cancer gene, rearrangements of which are disease defining in desmoplastic fibroblastoma, as shown in separate study^35^
Langerhans cell histiocytosis (CUH_0064)	Driver variant of Langerhans cell histiocytosis (*KLC1-RAF1*)
High-grade brain tumor (CUH_0082)	Tumor entity of high-grade embryonal tumors with *ATXN1–NUTM2D* fusion
B cell acute lymphoblastic leukemia (GOS_0011)	*PAX5* fusion partner in B cell acute lymphoblastic leukemia (*PAX5–NOC1*)
T cell lymphoblastic lymphoma (GOS_0066)	*NOTCH1* fusion partner in T cell lymphoblastic lymphoma (*NOTCH1–MTA1*)
ALCL (CUH_0054 and GOS_0036)	UV light signatures in childhood ALCL

Aside from specific variants, WGS analysis details somatic features of cancers more generally, including signatures of base substitutions that can be extracted from sequence context (that is, the base before and after a substitution). Signature analysis delivered by the central analysis pipeline (Methods and Supplementary Table 1) contributed to clinical impact in one case (CUH_0033, by providing diagnostic cues) and also delivered research hypotheses. For example, in two children (GOS_0036 and CUH_0054) with anaplastic large-cell lymphoma (ALCL), WGS showed a major contribution of ultraviolet (UV) light mutagenesis to the overall tumor mutation burden, which has not previously been reported in pediatric ALCL. This observation led us to speculate that UV light mutagenesis may, through the generation of additional driver events, underpin the otherwise unexplained increased recurrence risk that skin involvement imparts in childhood ALCL.

## Discussion

Our study provides a detailed assessment of the clinical utility of cancer WGS in children. Previous efforts in select patient groups, for example, children with high-risk cancers, have shown clinical benefits of WGS^12–18^. We now find that WGS also improves patient management when implemented into routine clinical practice. Of note, we found instances of practice-changing WGS findings in both the implementation and the routine testing phases and in children that mostly did not have high-risk disease (Table 3). This would support the proposition that WGS may be useful to all children with a neoplastic disorder^22^ and would argue against confining WGS to specific patient groups. The genetic findings that changed management here were varied. They did not feature targetable mutations, probably because treatment guidelines rarely advocate upfront mutation-targeted therapies over conventional treatment known to deliver excellent cure rates^33–36^. Unexpected germline cancer predispositions, however, featured prominently by providing opportunities for predisposition-directed therapy and potentially delivering lifelong benefits via cancer screening programs^37^.

We considered each child carefully within their specific clinical context to separate WGS findings that were disease relevant and potentially helpful, from those that actually changed practice, and that SOC testing would not have revealed. This approach enabled us to distill benefits that are likely to represent the added value of WGS in a real-world setting, across two operationally independent units that deploy extensive SOC molecular testing. In centers with less extensive SOC testing, the utility of WGS may be more pronounced. For example, most NHS pediatric hematology units do not use targeted RNA sequencing for fusion detection^38,39^ and would therefore benefit more from WGS. Conversely, it seems unlikely that more extensive SOC testing would negate all of the management-changing WGS benefits we saw unless an extensive testing regimen was administered to all children, namely, the combination of copy number arrays (tumor and germline), whole exome sequencing (tumor and germline) and unbiased RNA sequencing (tumor only).

A limitation of our national WGS pipeline has been its turnaround times that have not yet matched those of other efforts^9,15^. What constitutes a clinically meaningful turnaround time is debatable and varies by diagnosis and individual patient^9,40^. For example, within pediatric B cell leukemia, complete genomic testing should be returned before the end of induction chemotherapy (28 days) to enable adequate risk stratification^41^. By contrast, the genetic confirmation of acute promyelocytic leukemia has to occur within days of a provisional diagnosis^42^. We were able to prioritize patient groups where we foresaw particular benefit of WGS, but occasionally, results were not delivered fast enough to inform decision-making. A further limitation of our current genomic service is that RNA assessment is confined to panel sequencing for gene fusion detection and does not consider gene expression. However, previous studies indicate that additional clinical value can be gained from integrating cancer gene expression with variant data^9,16,17,23,43,44^.

We found that WGS accurately reproduced findings from every SOC molecular assay, suggesting that centralized WGS and variant calling could, in principle, replace all molecular assays deployed in this cohort. As sequencing cost decreases^45^, it is possible that WGS may provide opportunities for cost savings. However, this will have to be examined by detailed economic analyses that take into account factors specific to each healthcare setting. Beyond cost savings, an important consideration in pediatric oncological practice is tissue availability, as biopsy material is often limited, further supporting the consolidation of multiple SOC genomic assays into a single test. As large-scale cancer sequencing research efforts have concluded, the genomes of certain childhood cancers, their variants and ultra-rare entities remain relatively unexplored^46^. Our experience indicates that WGS will fill these gaps in our knowledge, an additional likely benefit from routine clinical WGS. Locally, WGS will enable clinicians to explore the cancer genomes of their patients, whereas nationally, NHS WGS data will gradually build a powerful resource for research, such as systematic genotype–phenotype correlations.

NHS England has chosen an unorthodox model for providing WGS to children with cancer, via a centralized sequencing and variant calling pipeline, independent of academic efforts, which aims to facilitate equitable access to molecularly informed cancer care. Advantages of this system include consistent data quality and localized decision-making within the specific clinical context of children. However, the system relies on local expertise for clinical variant interpretation, which may vary across units. Therefore, it is conceivable that our finding of utility may be specific to the NHS, and within England to our two units. On the other hand, genetic information forms the backbone of the treatment of most childhood cancers^47^. We would therefore suggest that our key finding of routine WGS delivering practice-changing benefits to children with suspected cancer is broadly applicable.

## Methods

### Ethics statement

Our study entitled ‘Assessing the Clinical Benefits of Whole-Genome Sequencing for Children with Neoplasms’ was approved by an NHS Research Ethics Committee (reference 22/WA/0281).

### Study design and participants

We carried out an observational study of patient cohorts from two English tertiary childhood cancer units, CUH and GOSH. Pediatric oncology services are distributed regionally; hence, patient selection was determined by the respective catchment areas of each unit—East Anglia for CUH and London and surrounding areas for GOSH—and was broadly representative of UK practice. At each center, there were two study periods: an implementation phase of nonconsecutive patients, selected at the discretion of their lead clinician based on the feasibility of appropriate sample acquisition and storage during the early establishment of the service, and a routine testing phase, aiming to offer WGS to every patient. The CUH program contributed data from children with solid tumors, and the GOSH cohort comprised children with hematological malignancies only. An NHS Research Ethics Committee (reference 22/WA/0281) approved this study, including the publication of relevant indirect identifiers. Participants and/or their legal guardians who did not provide consent to participate in research were excluded from analyses (Fig. 1). Three children enrolled in this study have been presented in case reports elsewhere (CUH_0006 (ref. ^48^), CUH_0034 (ref. ^49^) and CUH_0075 (ref. ^50^)), discussing specific clinical aspects. The genomic finding of one child (FOSL1 rearrangement in CUH_0048) formed the basis of a study establishing FOSL1 as a diagnostic marker^32^. One patient (CUH_0034) underwent WGS twice—both at initial diagnosis during the implementation phase and at relapse during the routine phase (Supplementary Table 1). One patient (GOS_0101) presenting in the study period underwent reanalysis of existing sequencing data that had been generated during the 100,000 Genomes Project.

### Workflow of WGS and analyses

Children with a suspected cancer diagnosis (including relapsed disease) were eligible for WGS, delivered by the NHS via a partnership with Genomics England (a company owned by the UK Department of Health) and Genomics Laboratory Hubs (GLHs) that serve as regional service centers (Fig. 2). WGS was carried out alongside SOC molecular assays unless there was limited tumor material, in which case existing SOC testing was prioritized. Patients were offered WGS in their local cancer unit (that is, CUH or GOSH) where biopsies and germline samples were obtained. Tumor DNA was derived from peripheral blood or bone marrow for liquid tumors and from fresh frozen tissue for solids, with local pathologists ascertaining adequate cellularity of neoplastic specimens. Germline DNA was extracted from disease-free peripheral blood, unaffected bone marrow or skin biopsy without fibroblast culture. The GLHs associated with each center prepared DNA that underwent WGS on Illumina’s short-read next-generation sequencing platform in a central national facility. The mean sequencing coverage was 108× for neoplasms and 43× for germline DNA. All classes of variants (substitutions, indels, copy number changes, rearrangements) were called centrally via the Genomics England Cancer Genome Analysis Pipeline (publicly available version: https://re-docs.genomicsengland.co.uk/cancer_2_28.pdf; the latest version is available on request at https://www.genomicsengland.co.uk/contact).

Additional analyses provided by the custom Genomics England pipeline included mutational signature analysis based on the catalog of mutational signatures as defined in COSMIC V2.2 (https://cancer.sanger.ac.uk/cosmic/signatures_v2)^51^ (Supplementary Table 1). In determining clinical utility, we referred to the signature analysis provided by the clinical pipeline. For the purposes of providing a data resource, we updated the signature analysis by performing de novo extraction using SigProfiler^52^ (COSMIC V3.3; https://cancer.sanger.ac.uk/cosmic/signatures)^51^ (Supplementary Table 1) on variant catalogs filtered against an additional panel of normal tissue sequences (to remove sequencing artifacts). The list of variants is available in Supplementary Table 2 (excluding one case of a hypermutated tumor with more than one million substitutions, CUH_0038, and one, GOS_0101, for which a variant call format file was not available for signature re-extraction). We provide results of both analyses in Supplementary Table 1.

The GEL WGS provisional report for each patient was returned to GLHs where clinical scientists interpreted findings in their clinical context. Treating clinicians had access to results as soon as they became available to facilitate timely decision-making. Results were also formally presented and discussed at genomics tumor advisory boards (GTABs) with a quorate representation from clinical scientists, pathologists, clinical geneticists and treating oncologists or hematologists.

To capture the meaningful turnaround time of results, in Supplementary Table 1, we present time from test request to GEL report availability (the point at which scientists and clinicians can access and use findings), as well as to GTAB, at which a finalized report is signed by a clinical scientist or pathologist.

### Impact analysis

As a basis for our analysis of impact, site investigators collected relevant clinical information, including WGS reports and results from other SOC molecular assays in real time. Every case was then retrospectively reviewed by at least four investigators, including the principal investigator at each study site, with consensus definitions of clinical impact as detailed in Table 1. We defined SOC as what was accepted local practice, stratified by diagnostic category. Some testing was mandatory (for example, FISH for leukemias) with others being discretionary based on expert review and clinical and pathological characteristics (Extended Data Table 1). Specific SOC assays used differed by treating center and disease type (Extended Data Table 2). We also judged SOC to include testing that should or would have been performed based on individual case detail to ensure that the unique impact of WGS was recorded (Fig. 3). For example, in two cases of rhabdomyosarcoma, WGS revealed treatment-changing *MYOD1* variants, but we judged that this should have been assessed in SOC (Supplementary Table 1) owing to specific histological features of the tumors.

We recorded whether WGS reproduced results from SOC molecular assays and whether it revealed any additional diagnostic, risk-defining, therapeutically targetable or pathogenic somatic or germline variants. Germline variant testing, in the form of targeted sequencing, arrays or karyotyping, was considered to be SOC in patients for whom it was performed in light of specific clinical features, family request and/or recommendation from a clinical geneticist. WGS data were not reanalyzed by local centers or investigators in light of SOC results. In assessing impact, we determined whether WGS altered patient management, aided diagnosis and/or provided therapeutic opportunities with each of these outcomes being delivered through specified routes (Table 2). We considered WGS to have altered practice only if the treatment-changing result had not been delivered by any alternate SOC test that had been, or should or would have been, requested. Through this approach, we aimed to show where added clinical value was exclusively attributed to WGS.

### Statistics and reproducibility

No statistical method was used to predetermine sample size. Randomization and blinding were not applicable to this observational case series. Clinical data were collated in Microsoft Excel version 16. Figures were generated in Adobe Illustrator version 27.9. R Studio Version 2023.06.3 and SigProfiler v3.3 (ref. ^52^) were used to carry out signature analysis.

### Reporting summary

Further information on research design is available in the Nature Portfolio Reporting Summary linked to this article.

## Online content

Any methods, additional references, Nature Portfolio reporting summaries, source data, extended data, supplementary information, acknowledgements, peer review information, details of author contributions and competing interests and statements of data and code availability are available at 10.1038/s41591-024-03056-w.

### Supplementary information

Reporting Summary

Supplementary Table 1 Per case summary of WGS data, SOC findings, and Impact analysis.

Supplementary Table 2 List of filtered substitutions used for de-novo signature extraction. CUH_0038 excluded (>1000000 substitutions), GOS_0101 excluded (VCF not available).

## Data Availability

De-identified patient-level information used for analysis is available in Supplementary Table 1. Variant data used for signature analysis are provided in Supplementary Table 2. Requests for raw sequencing data, variant calls, GEL signature analysis, quality metrics and a summary of findings submitted to Genomics Laboratory Hubs can be made via the Genomics England Research Environment, a secure cloud workspace. To access this workspace, researchers must apply for membership of the Genomics England Research Network via an academic institution as per the following steps: first, a signed participation agreement must be submitted by the institution to gecip-help@genomicsengland.co.uk. Then, following selection of an appropriate research domain, an online application should be submitted. Applications will be reviewed within ten working days, following which institutions must validate the researcher’s affiliation. If approved, access to the Genomics England Research Environment will be granted following successful completion of an online Information Governance Training module.

## Extended data

**Extended Data Table 1:** Standard of care testing strategy at diagnosis by site and diagnostic group

Categories			Somatic_genetic					Germline			Epigenetic	
Site	Disease Group	Disease Subgroup	Mutation-specific IHC	FISH	CN Array	Targeted DNA Sequencing	Targeted RNA Sequencing	Germline Karyotype	Germline CN Array	Germline Targeted DNA Sequencing	Methylation Array	Germline Methylation Studies
CUH	Bone or soft tissue sarcoma	Ewings	N	Y	N	N	Y	D	D	D	N	N
		Other	D	D	N	D	D	D	D	D	D	N
	Brain tumors	Medulloblastoma	N	Y	N	Y	D	D	D	D	Y	N
		Other	D	D	N	D	D	D	D	D	D	N
	Germ cell tumors		N	D	N	D	D	D	D	D	N	N
	Kidney tumors		D	D	N	D	D	D	D	D	N	D
	Neuroblastoma		D	Y	D*	D*	N	D	D	D	N	D
	Other	Neuroendocrine	N	D	N	D	D	D	D	D	D	N
		Histiocytoses	D	D	N	Y	D	D	D	D	N	N
		Carcinomas	D	N	N	D	D	D	D	D	N	N
		Non-sarcoma bone/soft tissue tumors	N	D	N	D	D	D	D	D	N	N
		Pleuropulmonary blastoma	N	N	N	Y	N	D	D	Y	N	N
		Malignant rhabdoid tumor	D	N	N	N	N	D	Y	Y	N	N
		Liver tumors	N	D	N	D	D	D	D	D	N	D
	Lymphoma - CUH		N	Y	N	N	N	D	D	D	N	N
GOS	Lymphoma - GOS		N	Y	N	D	D	D	D	D	N	N
	Lymphoblastic leukaemias		N	Y	Y†	Y	Y	D	D	D	N	N
	Myeloid leukaemias		N	Y	N	Y	Y^*#*^	D	D	D	N	N
	Bone marrow failures		N	Y	N	Y	D	D	D	Y	N	N

Y = Yes / should occur. N = No / would not usually be performed. D = Discretionary, as assessed in clinical and pathological context by the MDT. For example, if a germline variant was suggested by targeted sequencing (e.g. *DICER1* at VAF of 50%) or clinical features (e.g. bilateral renal tumours/dysmorphism) children would undergo germline testing for the relevant cancer predisposition. *Whilst discretionary, both copy number arrays and targeted seuqencing of the *ALK* gene are performed in most cases of neuroblastoma. Therefore, one may consider these two assays to be SOC. † = discretionary for T ALL and relapsed disease. ^#^Discretionary during implementation phase, but became routine durine study period for myeloid leukaemias excepting JMML and MLDS. Testing strategies differed in the case of relapsed disease, and were tailored on a case by case basis.

**Extended Data Table 2:** Standard of care assays used and their gene targets, listed by centre and disease group

Test	Panel Name	Site	Disease Group Used	Panel Link	Targets List
**Targeted DNA Panel**	RMH200 Solid tumor DNA NGS panel	CUH	Embryonal brain tumors | Kidney tumors | Sarcoma	https://rm-d8-live.s3.eu-west-1.amazonaws.com/d8live.royalmarsden.nhs.uk/s3fs-public/2022-05/RMH%20NGS%20Panels%20March%202022.pdf	ABL1; ABL2; ACACA; ACE; ACER1; ACKR3; ACSL6; ADD3; AFF1; AFF3; AFF4; AGR3; AHI1; AHRR; ALK; ANKRD28; AR; ARHGAP20; ARHGAP26; ARNT; ASPSCR1; ASTN2; ATF1; ATIC; ATP1B4; AUTS2; BACH2; BAG4; BAIAP2L1; BAZ2A; BCAS3; BCAS4; BCL10; BCL11A; BCL11B; BCL2; BCL2L1; BCL3; BCL6; BCL9; BCOR; BCR; BDNF; BICC1; BIRC3; BIRC6; BRAF; BRD1; BRD3; BRD4; BRWD3; BTBD18; BTG1; C11orf1; C11orf95; C2CD2L; C3orf27; CAMTA1; CAPRIN1; CARS; CASC5; CASP7; CBFA2T3; CBFB; CBL; CCAR2; CCDC28A; CCDC6; CCDC88C; CCNB1IP1; CCNB3; CCND1; CCND2; CCND3; CD74; CDH11; CDK5RAP2; CDK6; CDX1; CDX2; CEBPA; CEBPB; CEBPD; CEBPE; CEP170B; CEP85L; CHD6; CHIC2; CHMP2B; CHST11; CIC; CIITA; CLP1; CLTC; CLTCL1; CMKLR1; CNBP; CNOT2; CNTRL; COG5; COL1A1; COL1A2; COL6A3; COX6C; CPSF6; CRADD; CREB1; CREB3L1; CREB3L2; CREBBP; CRLF2; CRTC1; CSF1; CSF1R; CTDSP2; CTNNB1; CUX1; DAB2IP; DACH1; DACH2; DDIT3; DDX10; DDX20; DEK; DMRT1; DNAJB1; DPM1; DUSP22; DUX4; EBF1; EEFSEC; EGFR; EGR1; EGR2; EGR3; EGR4; EIF4A2; ELF4; ELK4; ELL; ELN; EML1; EML4; EP300; EP400; EPC1; EPOR; EPS15; ERBB3; ERC1; ERCC1; ERG; ERLIN2; ESR1; ETS1; ETV1; ETV4; ETV5; ETV6; EWSR1; EZR; FAM19A2; FCGR2B; FCRL4; FEN1; FEV; FGF8; FGFR1; FGFR1OP; FGFR1OP2; FGFR2; FGFR3; FGFR4; FHIT; FIP1L1; FLI1; FLNA; FLT3; FLT3LG; FNBP1; FOSB; FOSL1; FOXO1; FOXO4; FOXP1; FRK; FRYL; FUS; GAS5; GAS7; GATA1; GIT2; GLI1; GOSR1; GOT1; GPR128; GPR34; GRHPR; GRID1; GTF2I; H2AFX; HAS2; HEY1; HHEX; HIP1; HIPK1; HIST1H4I; HLF; HMGA2; HNF1A; HOXA10; HOXA11; HOXA13; HOXA9; HOXC11; HOXC13; HOXD11; HOXD13; HSP90AA1; ID4; IKZF1; IL2; IfL21R; IL3; INPP5D; IQCG; IRF2BP2; IRF4; IRS4; ITK; JAK1; JAK2; JAZF1; KANK1; KAT6A; KAT6B; KDM5A; KIAA1524; KIF5B; KMT2A; KPNB1; KSR1; LASP1; LCK; LCP1; LGR5; LHFP; LHX2; LHX4; LINC00598; LINC00982; LMBRD1; LMO1; LMO2; LNP1; LPP; LPXN; LRMP; LRRC37B; LTBP1; LYL1; MACROD1; MAF; MAFB; MALT1; MAML2; MAPRE1; MBNL1; MBTD1; MDS2; MEAF6; MECOM; MGEA5; MKL1; MKL2; MLF1; MLLT1; MLLT10; MLLT11; MLLT3; MLLT4; MLLT6; MN1; MNX1; MSI2; MSN; MUC1; MUTYH; MYB; MYBL1; MYC; MYH11; MYH9; MYO18A; MYO1F; NAB2; NAPA; NBEAP1; NBR1; NCOA1; NCOA2; NCOA3; NDE1; NF1; NFATC2; NFIB; NGF; NGFR; NIN; NIPBL; NKX2-5; NONO; NOTCH1; NPM1; NR4A3; NR6A1; NSD1; NT5C2; NTF3; NTF4; NTRK1; NTRK2; NTRK3; NUMA1; NUP107; NUP214; NUP98; NUTM1; NUTM2A; NUTM2B; OFD1; OLIG2; OLR1; OMD; P2RY8; PAPPA; PATZ1; PAX3; PAX5; PAX7; PAX8; PBX1; PCM1; PDE4DIP; PDGFB; PDGFRA; PDGFRB; PER1; PHF1; PHF23; PICALM; PIM1; PLAG1; PML; POM121; POU2AF1; POU5F1; PPAP2B; PPARG; PPARGC1A; PPFIBP1; PPP2R1B; PRCC; PRDM16; PRKACA; PRKAR1A; PRKG2; PRRX2; PSIP1; PSMD2; PTPRR; PVT1; RABEP1; RAD51B; RAF1; RANBP2; RAP1GDS1; RARA; RBM15; RBM6; RCOR1; RCSD1; RET; RHOH; RNF213; ROS1; RPL22; RPN1; RREB1; RRM1; RTEL1; RUNX1; RUNX1T1; SARNP; SEC31A; SEPT2; SEPT5; SEPT6; SEPT9; SERPINE1; SERPINF1; SET; SETBP1; SFPQ; SH3D19; SH3GL1; SIK3; SLC34A2; SLC45A3; SLCO1B3; SMAP1; SMARCA5; SMARCB1; SNHG5; SORBS2; SORT1; SP3; SPECC1; SPTBN1; SQSTM1; SRF; SRSF3; SS18; SS18L1; SSBP2; SSX1; SSX2; SSX4; ST6GAL1; STAT5B; STAT6; STRN; SUGP2; SUZ12; SYK; TACC1; TACC2; TACC3; TAF15; TAL1; TAL2; TAOK1; TBX15; TCF12; TCF3; TCL1A; TCTA; TEAD1; TEAD2; TEAD3; TEAD4; TEC; TENM1; TET1; TFE3; TFG; TFPT; TFRC; TGFBR3; THADA; THRAP3; TIRAP; TLX1; TLX3; TMPRSS2; TNFRSF17; TOP1; TOP2B; TP53BP1; TPM3; TPM4; TRHDE; TRIM24; TRIP11; TRPS1; USP16; USP42; USP6; VGLL3; WASF2; WDR18; WDR70; WHSC1; WHSC1L1; WSB1; WT1; WWTR1; XIAP; YAP1; YTHDF2; YWHAE; ZBTB16; ZC3H7A; ZC3H7B; ZFP64; ZFPM2; ZFYVE19; ZMIZ1; ZMYM2; ZMYND11; ZNF207; ZNF384; ZNF444; ZNF521; ZNF585B; ZNF687;
	Northeast and Yorkshire Targeted ALK Assay	CUH	Neuroblastoma	https://www.newcastlelaboratories.com/lab_service/laboratory-cancer-services/#yrk	Following *ALK* variants interrogated: c.3509T>C p.(Ile1170Thr) ; c.3509T>G p.(Ile1170Ser) ; c.3512T>A p.(Ile1171Asn) ; c.3512T>C p.(Ile1171Thr) ; c.3512T>G p.(Ile1171Ser) ; c.3520T>A p.(Phe1174Ile) ; c.3520T>C p.(Phe1174Leu) ; c.3520T>G p.(Phe1174Val) ; c.3521T>C p.(Phe1174Ser) ; c.3521T>G p.(Phe1174Cys) ; c.3521T>A p.(Phe1174Tyr) ; c.3522C>A p.(Phe1174Leu) ; c.3522C>G p.(Phe1174Leu) ; c.3718T>G p.(Leu1240Val) ; c.3718T>A p.(Leu1240Met) ; c.3509T>A p.(Ile1170Asn) ; c.3733T>G p.(Phe1245Val) ; c.3733T>C p.(Phe1245Leu) ; c.3734T>G p.(Phe1245Cys) ; c.3734T>C p.(Phe1245Ser) ; c.3734T>A p.(Phe1245Tyr) ; c.3735C>A p.(Phe1245Leu) ; c.3735C>G p.(Phe1245Leu) ; c.3824G>A p.(Arg1275Gln) ; c.3824G>T p.(Arg1275Leu) ; c.3824G>C p.(Arg1275Pro) ; c.3833A>C p.(Tyr1278Ser) ; c.3833A>G p.(Tyr1278Cys) ; c.3833A>T p.(Tyr1278Phe) ; c.3733T>A p.(Phe1245Ile) ;
	Ion AmpliSeq™ Cancer Hotspot Panel v2	CUH	All other disease types	https://www.thermofisher.com/order/catalog/product/4475346	ABL1; AKT1 ; ALK ; APC ; ATM ; BRAF; CDH1 ; CDKN2A ; CSF1R ; CTNNB1 ; EGFR ; ERBB2; ERBB4 ; EZH2 ; FBXW7 ; FGFR1; FGFR2 ; FGFR3 ; FLT3 ; GNA11 ; GNAS ; GNAQ ; HNF1A ; HRAS ; IDH1 ; IDH2 ; JAK2 ; JAK3 ; KDR ; KRAS; MET ; MLH1 ; MPL ; NOTCH1 ; NPM1 ; NRAS ; PDGFRA ; PIK3CA; PTEN ; PTPN11; RB1 ; RET; SMAD4 ; SMARCB1 ; SMO ; SRC ; STK11 ; TP53 ; VHL ;
	Royal Marsden HaemOnc NGS Panel	GOSH	Lymphoblastic leukaemia | Myeloid leukaemia | Bone marrow failure | Lymphoma | Other	https://rm-d8-live.s3.eu-west-1.amazonaws.com/d8live.royalmarsden.nhs.uk/s3fs-public/2022-05/RMH%20NGS%20Panels%20March%202022.pdf	ABCA1; ABL1; ACD; AKT1; ANKRD26; ARAF; ARID1A; ASXL1; ATM; ATR; ATRX; BCL11B; BCL2; BCL6; BCOR; BCORL1; BCR; BIRC3; BOD1L1; BRAF; BRCA1; BRCA2; BRCC3; BTK; CALR; CARD11; CBL; CBLB; CCND1; CCND2; CCND3; CD274; CD79B; CDKN2A; CDKN2B; CEBPA; CHEK2; CREBBP; CSF3R; CSNK1A1; CTC1; CTCF; CUX1; CXCR4; DCLRE1C; DDX41; DIS3; DKC1; DNAJC21; DNM2; DNMT3A; DNMT3B; DPYD; DUSP22; EED; ELANE; EP300; ERBB3; ETNK1; ETV6; EZH2; FAM46C; FANCL; FBXW7; FLT3; FOXO1; G6PC3; GATA1; GATA2; GFI1; GNAS; GNB1; GPRC5A; HAX1; HRAS; IDH1; IDH2; IKZF1; IL7R; IRF4; JAGN1; JAK1; JAK2; JAK3; KDM5A; KDM6A; KIT; KLF2; KMT2A; KMT2C; KMT2D; KMT2E; KRAS; LAMB4; LMO1; LMO2; MAP2K1; MAP3K1; MECOM; MEF2B; MET; MPL; MYB; MYC; MYCN; MYD88; NCOR1; NCOR2; NF1; NF2; NFE2; NOTCH1; NOTCH2; NPM1; NRAS; NRD1; NSD1; NT5C2; NTRK1; NTRK2; NTRK3; NUDT15; NUP98; PALB2; PAX5; PDGFRA; PDS5B; PHF6; PIGA; PIK3CA; PIK3CD; PIK3R1; PLCG2; PMS1; PMS2; PPM1D; PRPF8; PTEN; PTPN11; PTPRT; RAD21; RAD50; RAD51C; RAD51D; RHOA; RIT1; RPL10; RPL5; RUNX1; SAMD9; SAMD9L; SAMHD1; SBDS; SETBP1; SF1; SF3B1; SH2B3; SMC1A; SMC3; SRSF2; STAG2; STAT3; STAT5B; SUZ12; TAL1; TERC; TERT; TET1; TET2; TINF2; TP53; TPMT; TRAF2; U2AF1; U2AF2; USP7; VPS45; WAS; WT1; XPO1; XRCC2; ZRSR2;
**Germline DNA Panel**	Synnovis Cytopenia Gene Panel	GOSH	Bone marrow failure	https://www.synnovis.co.uk/our-tests/red-cell-gene-panel	ACD; ANKRD26; e; CECR1; (ADA2); CTC1; CXCR4; DKC1; DNAJC21; EFL1; ERCC6L2; G6PC3; GATA2; GFI1; HAX1; IKZF1; KIF23; KLF1; LAT; MPL; MYSM1; NBN; NHP2; NOP10; PARN; RTEL1; RUNX1; SAMD9; SAMD9L; SBDS; SRP72; TCN2; TERC; TERT; TINF2; USB1; VPS45; WRAP53;
**Targeted RNA Panel**	Illumina Trusight Pan Cancer Gene fusion panel	GOSH | CUH	Lymphoblastic leukaemia | Myeloid leukaemia | Bone marrow failure | Lymphoma | Sarcoma | Embryonal brain tumors	https://emea.support.illumina.com/downloads/trusight-rna-pan-cancer-target-regions-files.html	ABCC3; ABI1; ABL1; ABL2; ABLIM1; ACACA; ACE; ACER1; ACKR3; ACSBG1; ACSL3; ACSL6; ACVR1B; ACVR1C; ACVR2A; ADD3; ADM; AFF1; AFF3; AFF4; AGR3; AHCYL1; AHI1; AHR; AHRR; AIP; AK2; AK5; AKAP12; AKAP6; AKAP9; AKR1C3; AKT1; AKT2; AKT3; ALDH1A1; ALDH2; ALDOC; ALK; AMER1; AMH; ANGPT1; ANKRD28; ANLN; APC; APH1A; APLP2; APOD; AR; ARAF; ARFRP1; ARHGAP20; ARHGAP26; ARHGEF12; ARHGEF7; ARID1A; ARID2; ARIH2; ARNT; ARRDC4; ASMTL; ASPH; ASPSCR1; ASTN2; ASXL1; ATF1; ATF3; ATG13; ATG5; ATIC; ATL1; ATM; ATP1B4; ATP8A2; ATR; ATRNL1; ATRX; AURKA; AURKB; AUTS2; AXIN1; AXL; BACH1; BACH2; BAG4; BAIAP2L1; BAP1; BARD1; BAX; BAZ2A; BCAS3; BCAS4; BCL10; BCL11A; BCL11B; BCL2; BCL2A1; BCL2L1; BCL2L2; BCL3; BCL6; BCL7A; BCL9; BCOR; BCORL1; BCR; BDNF; BHLHE22; BICC1; BIN1; BIRC3; BIRC6; BLM; BMP4; BMPR1A; BRAF; BRCA1; BRCA2; BRD1; BRD3; BRD4; BRIP1; BRSK1; BRWD3; BTBD18; BTG1; BTG2; BTK; BTLA; BUB1B; C11orf1; C11orf30; C11orf54; C11orf95; C2CD2L; C2orf44; C3orf27; CACNA1F; CACNA1G; CACNA2D3; CAD; CALR; CAMK2A; CAMK2B; CAMK2G; CAMTA1; CANT1; CAPRIN1; CAPZB; CARD11; CARM1; CARS; CASC5; CASP3; CASP7; CASP8; CAV1; CBFA2T3; CBFB; CBL; CBLB; CBLC; CCAR2; CCDC28A; CCDC6; CCDC88C; CCK; CCL2; CCNA2; CCNB1IP1; CCNB3; CCND1; CCND2; CCND3; CCNE1; CCNG1; CCT6B; CD19; CD22; CD274; CD28; CD36; CD44; CD58; CD70; CD74; CD79A; CD79B; CD8A; CDC14A; CDC14B; CDC25A; CDC25C; CDC42; CDC73; CDH1; CDH11; CDK1; CDK12; CDK2; CDK4; CDK5RAP2; CDK6; CDK7; CDK8; CDK9; CDKL5; CDKN1A; CDKN1B; CDKN1C; CDKN2A; CDKN2B; CDKN2C; CDKN2D; CDX1; CDX2; CEBPA; CEBPB; CEBPD; CEBPE; CENPF; CENPU; CEP170B; CEP57; CEP85L; CHCHD7; CHD2; CHD6; CHEK1; CHEK2; CHIC2; CHL1; CHMP2B; CHN1; CHST11; CHUK; CIC; CIITA; CIRH1A; CIT; CKB; CKS1B; CLP1; CLTA; CLTC; CLTCL1; CMKLR1; CNBP; CNOT2; CNTN1; CNTRL; COG5; COL11A1; COL1A1; COL1A2; COL3A1; COL6A3; COL9A3; COMMD1; COX6C; CPNE1; CPS1; CPSF6; CRADD; CREB1; CREB3L1; CREB3L2; CREBBP; CRKL; CRLF2; CRTC1; CRTC3; CSF1; CSF1R; CSF3; CSF3R; CSNK1G2; CSNK2A1; CTCF; CTDSP2; CTLA4; CTNNA1; CTNNB1; CTNND2; CTRB1; CTSA; CUX1; CXCL8; CXCR4; CXXC4; CYFIP2; CYLD; CYP1B1; CYP2C19; DAB2IP; DACH1; DACH2; DAXX; DCLK2; DCN; DDB2; DDIT3; DDR2; DDX10; DDX20; DDX39B; DDX3X; DDX5; DDX6; DEK; DGKB; DGKI; DGKZ; DICER1; DIRAS3; DIS3L2; DKK1; DKK2; DKK4; DLEC1; DLL1; DLL3; DLL4; DMRT1; DMRTA2; DNAJB1; DNM1; DNM2; DNM3; DNMT1; DNMT3A; DOCK1; DOT1L; DPM1; DPYD; DST; DTX1; DTX4; DUSP2; DUSP22; DUSP26; DUSP9; DUX4; E2F1; EBF1; ECT2L; EDIL3; EDNRB; EED; EEFSEC; EGF; EGFR; EGR1; EGR2; EGR3; EGR4; EIF4A2; EIF4E; ELF4; ELK4; ELL; ELN; ELOVL2; ELP2; EML1; EML4; ENPP2; EP300; EP400; EPC1; EPCAM; EPHA10; EPHA2; EPHA3; EPHA5; EPHA7; EPHB1; EPHB6; EPO; EPOR; EPS15; ERBB2; ERBB3; ERBB4; ERC1; ERCC1; ERCC2; ERCC3; ERCC4; ERCC5; ERCC6; ERG; ERLIN2; ESR1; ETS1; ETS2; ETV1; ETV4; ETV5; ETV6; EWSR1; EXOSC6; EXT1; EXT2; EYA1; EYA2; EZH2; EZR; FAF1; FAM127C; FAM19A2; FAM19A5; FAM46C; FAM64A; FANCA; FANCB; FANCC; FANCD2; FANCE; FANCF; FANCG; FANCI; FANCL; FANCM; FAS; FASLG; FBN2; FBXO11; FBXO31; FBXW7; FCGBP; FCGR2B; FCRL4; FEN1; FEV; FGF1; FGF10; FGF13; FGF14; FGF19; FGF2; FGF23; FGF3; FGF4; FGF6; FGF8; FGF9; FGFR1; FGFR1OP; FGFR1OP2; FGFR2; FGFR3; FGFR4; FH; FHIT; FHL2; FIGF; FIP1L1; FLCN; FLI1; FLNA; FLNC; FLT1; FLT3; FLT3LG; FLT4; FLYWCH1; FNBP1; FOS; FOSB; FOSL1; FOXL2; FOXO1; FOXO3; FOXO4; FOXP1; FRK; FRMPD4; FRS2; FRYL; FSTL3; FUS; FUT1; FZD10; FZD2; FZD3; FZD6; FZD7; FZD8; GAB1; GABRG2; GADD45B; GANAB; GAS1; GAS5; GAS7; GATA1; GATA2; GATA3; GATA6; GBP2; GDF6; GFAP; GHR; GID4; GIT2; GLI1; GLI3; GMPS; GNA11; GNA12; GNA13; GNAI1; GNAQ; GNAS; GNG4; GOLGA5; GOPC; GOSR1; GOT1; GPC3; GPHN; GPR124; GPR128; GPR34; GRB10; GRB2; GRHPR; GRID1; GRIN2A; GRIN2B; GRM1; GRM3; GSK3B; GSN; GSTT1; GTF2I; GTSE1; H2AFX; H3F3A; HAS2; HDAC1; HDAC2; HDAC3; HDAC4; HDAC5; HDAC6; HDAC7; HECW1; HEPH; HERPUD1; HES1; HES5; HEY1; HGF; HHEX; HIF1A; HIP1; HIPK1; HIPK2; HIST1H1C; HIST1H1D; HIST1H1E; HIST1H2AC; HIST1H2AG; HIST1H2AL; HIST1H2AM; HIST1H2BC; HIST1H2BJ; HIST1H2BK; HIST1H2BO; HIST1H3B; HIST1H4I; HLF; HMGA1; HMGA2; HMGB1; HMGN2P46; HNF1A; HNRNPA2B1; HOOK3; HOXA10; HOXA11; HOXA13; HOXA3; HOXA9; HOXC11; HOXC13; HOXD11; HOXD13; HOXD9; HRAS; HSP90AA1; HSP90AB1; HSPA1A; HSPA2; HSPA4; HSPA5; HTRA1; HUWE1; IBSP; ICAM1; ICK; ID1; ID3; ID4; IDH1; IDH2; IFNG; IFRD1; IGF1; IGF1R; IGFBP2; IGFBP3; IKBKB; IKBKE; IKZF1; IKZF2; IKZF3; IL12RB2; IL13; IL13RA2; IL15; IL1B; IL1R1; IL1RAP; IL2; IL21R; IL2RA; IL3; IL6; IL7R; INHBA; INPP4A; INPP4B; INPP5A; INPP5D; IQCG; IRF1; IRF2BP2; IRF4; IRF8; IRS1; IRS2; IRS4; ITGA5; ITGA7; ITGA8; ITGAV; ITGB3; ITK; ITPKA; JAG2; JAK1; JAK2; JAK3; JARID2; JAZF1; JUN; KALRN; KANK1; KAT2B; KAT6A; KAT6B; KCNB1; KDM1A; KDM2B; KDM4C; KDM5A; KDM5C; KDM6A; KDR; KDSR; KEAP1; KIAA0232; KIAA1524; KIAA1549; KIAA1598; KIF5B; KIT; KLF4; KLHL6; KLK2; KLK7; KMT2A; KMT2B; KMT2C; KMT2D; KPNB1; KRAS; KSR1; KTN1; LAMA1; LAMA5; LAMP2; LASP1; LCK; LCP1; LEF1; LEFTY2; LFNG; LGALS3; LGR5; LHFP; LHX2; LHX4; LIFR; LINC00598; LINC00982; LINGO2; LMBRD1; LMO1; LMO2; LMO7; LNP1; LOX; LPAR1; LPP; LPXN; LRIG3; LRMP; LRP1B; LRP5; LRPPRC; LRRC37B; LRRC59; LRRC7; LRRK2; LTBP1; LYL1; LYN; MACROD1; MAD2L1; MADD; MAF; MAFB; MAGED1; MAGEE1; MALAT1; MALT1; MAML1; MAML2; MAP2; MAP2K1; MAP2K2; MAP2K3; MAP2K4; MAP2K5; MAP2K6; MAP2K7; MAP3K1; MAP3K14; MAP3K6; MAP3K7; MAPK1; MAPK3; MAPK8; MAPK8IP2; MAPK9; MAPRE1; MATK; MAX; MB21D2; MBNL1; MBTD1; MCL1; MDC1; MDH1; MDM2; MDM4; MDS2; MEAF6; MECOM; MED12; MEF2B; MEF2C; MEF2D; MELK; MEN1; MET; METTL18; METTL7B; MFNG; MGEA5; MGMT; MIB1; MIPOL1; MITF; MKI67; MKL1; MKL2; MLF1; MLH1; MLLT1; MLLT10; MLLT11; MLLT3; MLLT4; MLLT6; MMP7; MMP9; MN1; MNAT1; MNX1; MPL; MRE11A; MSH2; MSH3; MSH6; MSI2; MSN; MTCP1; MTOR; MTUS2; MUC1; MUTYH; MYB; MYBL1; MYC; MYCL; MYCN; MYD88; MYH11; MYH9; MYO18A; MYO1F; NAB2; NACA; NAPA; NAV3; NBEAP1; NBN; NBR1; NCAM1; NCKIPSD; NCOA1; NCOA2; NCOA3; NCOA4; NCOR2; NCSTN; NDC80; NDE1; NDRG1; NDUFAF1; NEDD4; NEURL1; NF1; NF2; NFATC1; NFATC2; NFE2L2; NFIB; NFKB1; NFKB2; NFKBIA; NGF; NGFR; NIN; NIPBL; NKX2-1; NKX2-5; NOD1; NODAL; NONO; NOS3; NOTCH1; NOTCH2; NOTCH3; NOTCH4; NPM1; NPM2; NR3C1; NR4A3; NR6A1; NRAS; NSD1; NT5C2; NTF3; NTF4; NTRK1; NTRK2; NTRK3; NUMA1; NUP107; NUP214; NUP93; NUP98; NUTM1; NUTM2A; NUTM2B; OFD1; OLIG1; OLIG2; OLR1; OMD; P2RY8; PAFAH1B2; PAG1; PAK1; PAK3; PAK6; PAK7; PALB2; PAPPA; PASK; PATZ1; PAX3; PAX5; PAX7; PAX8; PBRM1; PBX1; PC; PCBP1; PCLO; PCM1; PCNA; PCSK7; PDCD1; PDCD11; PDCD1LG2; PDE4DIP; PDGFA; PDGFB; PDGFD; PDGFRA; PDGFRB; PDK1; PEG3; PER1; PFDN5; PHB; PHF1; PHF23; PHF6; PHOX2B; PI4KA; PICALM; PIK3CA; PIK3CB; PIK3CD; PIK3CG; PIK3R1; PIK3R2; PIM1; PKM; PLA2G2A; PLA2G5; PLAG1; PLAT; PLAU; PLCB1; PLCB4; PLCG1; PLCG2; PLEKHM2; PML; PMS1; PMS2; POFUT1; POLD1; POLD4; POLR2H; POM121; POMGNT1; POSTN; POT1; POU2AF1; POU5F1; PPAP2B; PPARG; PPARGC1A; PPFIA2; PPFIBP1; PPM1D; PPP1CB; PPP1R13B; PPP1R13L; PPP2CB; PPP2R1A; PPP2R1B; PPP2R2B; PPP2R4; PPP3CA; PPP3CB; PPP3CC; PPP3R1; PPP3R2; PPP4C; PQLC3; PRCC; PRDM1; PRDM16; PRDM7; PRF1; PRG2; PRICKLE1; PRKACA; PRKACG; PRKAR1A; PRKCA; PRKCB; PRKCD; PRKCG; PRKDC; PRKG2; PRMT1; PRMT8; PROM1; PRRX1; PRRX2; PRSS8; PSD3; PSEN1; PSIP1; PSMD2; PTBP1; PTCH1; PTCRA; PTEN; PTGS2; PTK2; PTK2B; PTK7; PTPN11; PTPN2; PTPN6; PTPRA; PTPRK; PTPRO; PTPRR; PTTG1; PVT1; RABEP1; RAC1; RAC2; RAC3; RAD21; RAD50; RAD51; RAD51B; RAD51C; RAD51D; RAD52; RAF1; RALGDS; RANBP17; RANBP2; RAP1GDS1; RARA; RASAL1; RASGEF1A; RASGRF1; RASGRF2; RASGRP1; RB1; RBM15; RBM6; RCHY1; RCOR1; RCSD1; RECQL4; REEP3; RELA; RELN; RERG; RET; RGS7; RHBDF2; RHOA; RHOD; RHOH; RICTOR; RLTPR; RMI2; RNF213; RNF43; ROBO1; ROBO2; ROS1; RPA3; RPL22; RPN1; RPN2; RPS21; RPS6KA1; RPS6KA2; RPS6KA3; RPTOR; RREB1; RRM1; RRM2B; RTEL1; RTN3; RUNX1; RUNX1T1; RUNX2; RYR3; S1PR2; SARNP; SBDS; SCN8A; SDC4; SDHA; SDHAF2; SDHB; SDHC; SDHD; SEC31A; SERP2; SERPINE1; SERPINF1; SET; SETBP1; SETD2; SETD7; SF3B1; SFPQ; SFRP2; SFRP4; SGK1; SGPP2; SH2D5; SH3BP1; SH3D19; SH3GL1; SH3GL2; SHC1; SHC2; SIK3; SIN3A; SIRT1; SKP2; SLC1A2; SLC34A2; SLC45A3; SLC7A5; SLCO1B3; SLX4; SMAD2; SMAD3; SMAD4; SMAD6; SMAP1; SMARCA1; SMARCA4; SMARCA5; SMARCB1; SMC1A; SMC3; SMO; SNAPC3; SNCG; SNHG5; SNW1; SNX29; SNX9; SOCS1; SOCS2; SOCS3; SOD2; SORBS2; SORT1; SOS1; SOX10; SOX11; SOX2; SP1; SP3; SPECC1; SPEN; SPOP; SPP1; SPRY2; SPRY4; SPTAN1; SPTBN1; SQSTM1; SRC; SRF; SRGAP3; SRRM3; SRSF2; SRSF3; SS18; SS18L1; SSBP2; SSX1; SSX2; SSX4; ST6GAL1; STAG2; STAT1; STAT3; STAT4; STAT5A; STAT5B; STAT6; STIL; STK11; STL; STRN; STX5; STYK1; SUFU; SUGP2; SULF1; SUV39H2; SUZ12; SYK; SYP; TACC1; TACC2; TACC3; TAF1; TAF15; TAL1; TAL2; TAOK1; TBL1XR1; TBX15; TCEA1; TCF12; TCF3; TCF7L2; TCL1A; TCL6; TCTA; TEAD1; TEAD2; TEAD3; TEAD4; TEC; TENM1; TERF1; TERF2; TERT; TET1; TET2; TFAP2A; TFDP1; TFE3; TFEB; TFG; TFPT; TFRC; TGFB2; TGFB3; TGFBI; TGFBR2; TGFBR3; THADA; THBS1; THRAP3; TIAM1; TIRAP; TLL2; TLR4; TLX1; TLX3; TMEM127; TMEM230; TMEM30A; TMPRSS2; TNC; TNF; TNFAIP3; TNFRSF10B; TNFRSF10D; TNFRSF11A; TNFRSF14; TNFRSF17; TNFRSF6B; TOP1; TOP2A; TOP2B; TP53; TP53BP1; TP63; TP73; TPD52L2; TPM3; TPM4; TPO; TPR; TRAF2; TRAF3; TRAF5; TRHDE; TRIM24; TRIM27; TRIM33; TRIP11; TRPS1; TSC1; TSC2; TSHR; TTK; TTL; TUSC3; TYK2; TYMS; U2AF1; U2AF2; UBE2B; UBE2C; UFC1; UFM1; USP16; USP42; USP5; USP6; USP7; VCAM1; VEGFA; VEGFC; VGLL3; VHL; VTI1A; WASF2; WDFY3; WDR1; WDR18; WDR70; WDR90; WEE1; WHSC1; WHSC1L1; WIF1; WISP3; WNT10A; WNT10B; WNT11; WNT16; WNT2B; WNT3; WNT4; WNT5B; WNT6; WNT7B; WNT8B; WRN; WSB1; WT1; WWOX; WWTR1; XBP1; XIAP; XKR3; XPA; XPC; XPO1; XRCC6; YAP1; YPEL5; YTHDF2; YWHAE; YY1AP1; ZBTB16; ZC3H7A; ZC3H7B; ZFP64; ZFPM2; ZFYVE19; ZIC2; ZMIZ1; ZMYM2; ZMYM3; ZMYND11; ZNF207; ZNF217; ZNF24; ZNF331; ZNF384; ZNF444; ZNF521; ZNF585B; ZNF687; ZNF703; ZRSR2; NCAM1; PPARGC1A; TFRC;
**Copy Number Array**	Whole genome Infinium CytoSNP-850K v*1.2* Illumina BeadChip 850K	GOSH | CUH	Lymphoblastic leukaemia | Neuroblastoma	https://emea.illumina.com/products/by-type/clinical-research-products/infinium-cytosnp-850k.html	NA

## Code availability

No custom code was used in the production of this paper.
