# Supplementary material for: Benefits for children with suspected cancer from routine whole-genome sequencing
Source: Nat Med. 2024 Jul 2;30(7):1905–12. doi: 10.1038/s41591-024-03056-w (PMC11271414; doi:10.1038/s41591-024-03056-w)
Supplement: Supplementary file 1 — Reporting Summary [file 41591_2024_3056_MOESM1_ESM.pdf]

Corresponding author(s): Professor Sam Behjati

Last updated by author(s): 19/04/2024

Reporting Summary

Nature Portfolio wishes to improve the reproducibility of the work that we publish. This form provides structure for consistency and transparency in reporting. For further information on Nature Portfolio policies, see our Editorial Policies and the Editorial Policy Checklist.

Statistics

For all statistical analyses, confirm that the following items are present in the figure legend, table legend, main text, or Methods section.

n/a	Confirmed	
☐	☒	The exact sample size (*n*) for each experimental group/condition, given as a discrete number and unit of measurement
☐	☒	A statement on whether measurements were taken from distinct samples or whether the same sample was measured repeatedly
☒	☐	The statistical test(s) used AND whether they are one- or two-sided<br>*Only common tests should be described solely by name; describe more complex techniques in the Methods section.*
☒	☐	A description of all covariates tested
☒	☐	A description of any assumptions or corrections, such as tests of normality and adjustment for multiple comparisons
☒	☐	A full description of the statistical parameters including central tendency (e.g. means) or other basic estimates (e.g. regression coefficient) AND variation (e.g. standard deviation) or associated estimates of uncertainty (e.g. confidence intervals)
☒	☐	For null hypothesis testing, the test statistic (e.g. *F*, *t*, *r*) with confidence intervals, effect sizes, degrees of freedom and *P* value noted<br>*Give P values as exact values whenever suitable.*
☒	☐	For Bayesian analysis, information on the choice of priors and Markov chain Monte Carlo settings
☒	☐	For hierarchical and complex designs, identification of the appropriate level for tests and full reporting of outcomes
☒	☐	Estimates of effect sizes (e.g. Cohen's *d*, Pearson's *r*), indicating how they were calculated

Our web collection on statistics for biologists contains articles on many of the points above.

Software and code

Policy information about availability of computer code

Data collection	Microsoft Excel Version 16
Data analysis	Figures were generated in Adobe Illustrator version 27.9. Signature analysis was performed using R Studio Version 2023.06.3 and SigProfiler v3.3

For manuscripts utilizing custom algorithms or software that are central to the research but not yet described in published literature, software must be made available to editors and reviewers. We strongly encourage code deposition in a community repository (e.g. GitHub). See the Nature Portfolio guidelines for submitting code & software for further information.

Data

Policy information about availability of data

All manuscripts must include a data availability statement. This statement should provide the following information, where applicable:

- Accession codes, unique identifiers, or web links for publicly available datasets
- A description of any restrictions on data availability
- For clinical datasets or third party data, please ensure that the statement adheres to our policy

De-identified patient level information used for analysis is available in Extended Data Table 1. Variant data used for signature analysis is provided in Extended Data Table 4. Requests for raw sequencing data, variant calls, GEL signature analysis, quality metrics and a summary of findings submitted to Genomics Laboratory Hubs can be made via the Genomics England Research Environment, a secure cloud workspace. To access this workspace, researchers must apply for membership of the

Genomics England Research Network via an academic institution as per the following steps: first, a signed participation agreement must be submitted by the institution to gecip-help@genomicsengland.co.uk. Then, following selection of an appropriate research domain, an online application should be submitted. Applications will be reviewed within ten working days, following which institutions must validate the researcher's affiliation. If approved, access to the Research Environment will be granted following successful completion of an online Information Governance Training module.

Research involving human participants, their data, or biological material

Policy information about studies with human participants or human data. See also policy information about sex, gender (identity/presentation), and sexual orientation and race, ethnicity and racism.

Reporting on sex and gender	Our findings applied to both sexes. We do not report individual sex and gender to aid de-identification.
Reporting on race, ethnicity, or other socially relevant groupings	We did not analyse social groupings, including race or ethnicity, in this study.
Population characteristics	No covariate features were relevant to our analysis
Recruitment	Patients come from geographically defined populations in England that each of the centers serve. The majority of presenting patients were recruited, with exclusions defined in Figure 1. Exclusions were primarily determined by sample availability or because of disease-specific factors.
Ethics oversight	Our study, entitled "Assessing the clinical benefits of whole genome sequencing for children with neoplasms", was approved by an NHS Research Ethics Committee (reference 22/WA/0281).

Note that full information on the approval of the study protocol must also be provided in the manuscript.

Field-specific reporting

Please select the one below that is the best fit for your research. If you are not sure, read the appropriate sections before making your selection.

☒ Life sciences ☐ Behavioural & social sciences ☐ Ecological, evolutionary & environmental sciences

For a reference copy of the document with all sections, see nature.com/documents/nr-reporting-summary-flat.pdf

Life sciences study design

All studies must disclose on these points even when the disclosure is negative.

Sample size	Not applicable to this observational case series.
Data exclusions	Nil
Replication	This does not apply to our study/data
Randomization	Not applicable to this observational case series.
Blinding	Not applicable to this observational case series.

Reporting for specific materials, systems and methods

We require information from authors about some types of materials, experimental systems and methods used in many studies. Here, indicate whether each material, system or method listed is relevant to your study. If you are not sure if a list item applies to your research, read the appropriate section before selecting a response.

Materials & experimental systems

n/a	Involved in the study	
☒	☐	Antibodies
☒	☐	Eukaryotic cell lines
☒	☐	Palaeontology and archaeology
☒	☐	Animals and other organisms
☐	☒	Clinical data
☒	☐	Dual use research of concern
☒	☐	Plants

Methods

n/a	Involved in the study	
☒	☐	ChIP-seq
☒	☐	Flow cytometry
☒	☐	MRI-based neuroimaging

Clinical data

Policy information about clinical studies

All manuscripts should comply with the ICMJE guidelines for publication of clinical research and a completed CONSORT checklist must be included with all submissions.

Clinical trial registration	Not relevant as this study was not an interventional trial.
Study protocol	Not relevant as this study was not an interventional trial.
Data collection	Recruitment started in January 2021, with data collection until December 2022. Data analysis was carried out from January 2021 until October 2023.
Outcomes	Impact of whole genome sequencing was assigned based on agreed definitions outlined in Table 1.

Plants

Seed stocks	NA (error in form)
Novel plant genotypes	NA (error in form)
Authentication	NA (error in form)
